# A unique case of glucagonoma with atypical necrolytic migratory erythema

**DOI:** 10.1016/j.jdcr.2025.06.009

**Published:** 2025-06-19

**Authors:** Robert J. Vanaria, Ide K. Kafexhiu, Asena Bahce-Altuntas, Nooshin Brinster, Alejandro Garcia, Colette M. Knight, Mariela Mitre

**Affiliations:** a Hackensack Meridian School of Medicine, Nutley, New Jersey; b CUNY School of Medicine, New York, New York; c Rheumatology Division, Department of Internal Medicine, Hackensack University Medical Center, Hackensack, New Jersey; d Bridge Dermpath, Tarrytown, New York; e Endocrine Division, Department of Internal Medicine, Hackensack University Medical Center, Hackensack, New Jersey; f Dermatology Division, Department of Internal Medicine, Hackensack University Medical Center, Hackensack, New Jersey

**Keywords:** cutaneous oncology, dermatitis, glucagonoma, necrolytic migratory erythema, paraneoplastic

## Case description

**Fig 1:**
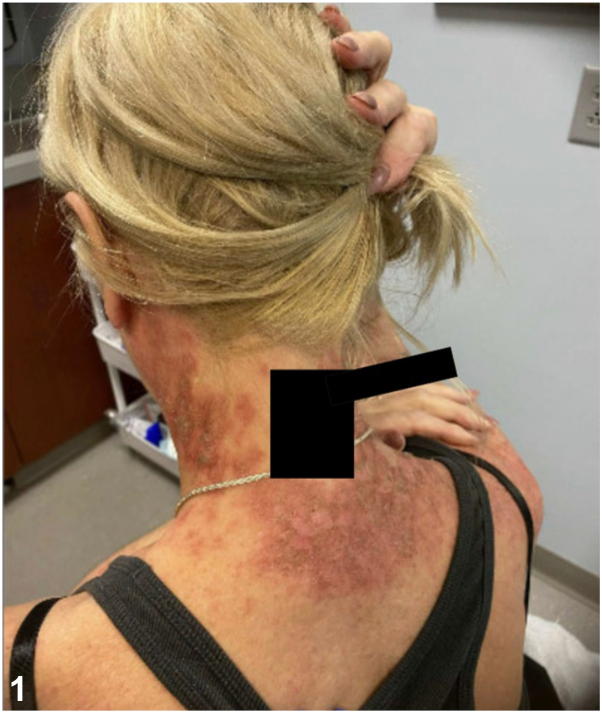

**Fig 2:**
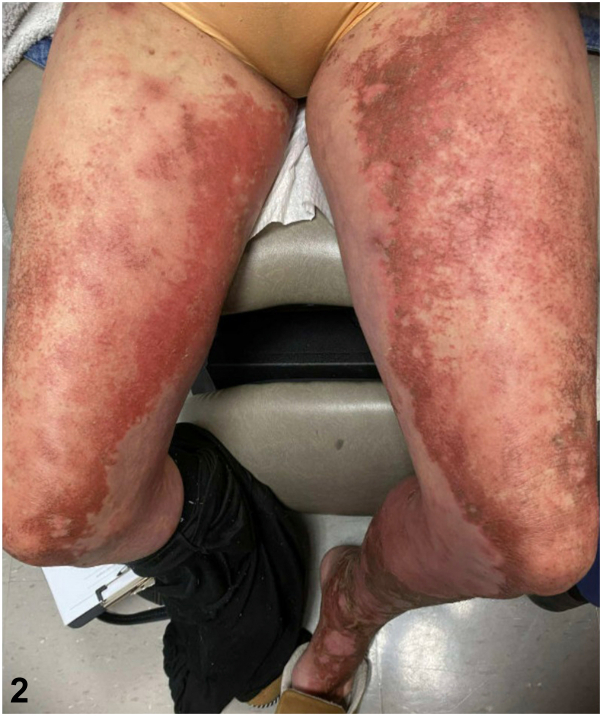

A 54-year-old woman with a medical history of ulcerative colitis (UC) and gastroesophageal reflux disease presented to the Hackensack University Medical Center Dermatology clinic in December 2023 for a severe rash. The rash began on her feet in 2022, spreading to her neck, back, face, and legs (Figs 1 and 2). She was initially treated with cephalexin and hospitalized for presumed Stevens-Johnson syndrome, but the rash recurred after corticosteroid tapering. A 2023 skin biopsy revealed spongiotic dermatitis with conspicuous keratinocyte necrosis, leading to the treatment with dupilumab and topical steroids for presumed atopic dermatitis, although symptoms persisted. Suspecting an extraintestinal manifestation of UC, her rheumatologist initiated adalimumab. She was later hospitalized for a deep vein thrombosis and pulmonary embolism a month after a total hip arthroplasty and started on anticoagulation. Further workup, including a positive antinuclear antibody test (1:320), was inconclusive for autoimmune disease. Repeat biopsy in November 2023 again showed spongiotic dermatitis, and a computed tomography (CT) scan ruled out malignancy.

**Fig 3:**
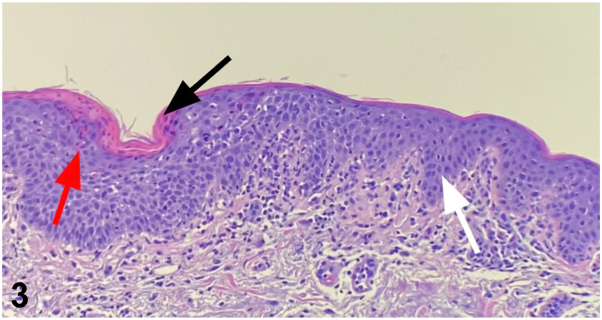

On presentation to our dermatology clinic, the patient had an extensive, well-demarcated, serpiginous rash with eczematous patches and plaques with crusting. There was no mucosal involvement or systemic symptoms such as joint pain or gastrointestinal distress. Broad shave biopsy was performed to rule out cutaneous T-cell lymphoma, dermatomyositis, necrolytic migratory erythema (NME), and necrolytic acral erythema (NAE). She was prescribed triamcinolone 0.1% and hydroxyzine 25 mg for pruritus. Biopsy findings (Fig 3) of psoriasiform dermatitis with conspicuous keratinocyte necrosis raised concerns for nutritional deficiency, NME, or NAE.

**What is the diagnosis?**

**A.** Cutaneous lupus erythematosus
**B.** Cutaneous T-cell lymphoma
**C.** IBD-related dermatosis
**D.** Glucagonoma
**E.** Amyopathic dermatomyositis

**Answers:**

**A.** Cutaneous lupus erythematosus – Incorrect. This typically shows interface dermatitis with basal layer damage and mucin deposition, not psoriasiform changes with keratinocyte necrosis.
**B.** Cutaneous T-cell lymphoma – Incorrect. CTCL usually presents with epidermotropism of atypical lymphocytes and Pautrier microabscesses, which were not seen on biopsy.
**C.** IBD-related dermatosis – Incorrect. Common IBD-associated skin findings like pyoderma gangrenosum or erythema nodosum do not match this patient's psoriasiform histology and chronic serpiginous eruption.
**D.** Glucagonoma – Correct. The characteristic psoriasiform histology pattern with conspicuous keratinocyte necrosis and lack of response to biologics suggests necrolytic migratory erythema from an existing glucagonoma. Other options are inconsistent with chronicity and biopsy findings.
**E.** Amyopathic dermatomyositis – Incorrect – This diagnosis is unlikely given the absence of muscle involvement, systemic symptoms, and lack of classic histologic features such as interface dermatitis or mucin.

**Clinical question: Which skin biopsy histological feature should raise suspicion of a glucagonoma or other nutritional deficiency?**

**A.** Neutrophilic inflammation with leukocytoclasia
**B.** Necrobiotic granulomas surrounded by palisaded lymphocytes and histiocytes
**C.** Conspicuous keratinocyte necrosis
**D.** Interface dermatitis with Civatte bodies
**E.** Perivascular dermatitis with plasma cells

**Correct answer: (C)** Conspicuous keratinocyte necrosis.

While the presence of spongiotic and then psoriasiform dermatitis in the biopsies is nonspecific, conspicuous keratinocyte necrosis is a feature that should raise the suspicion for a nutritional deficiency, NME, or NAE.

**Fig 4:**
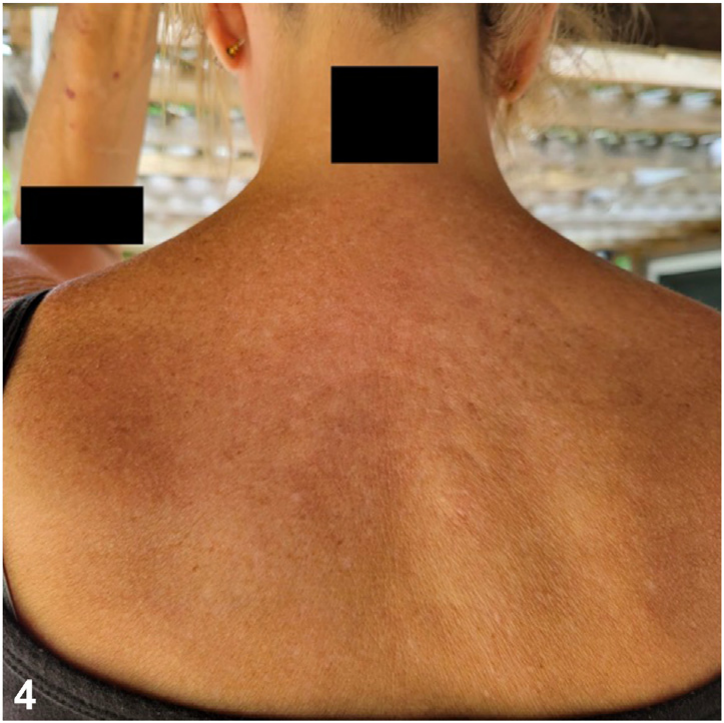

**Fig 5:**
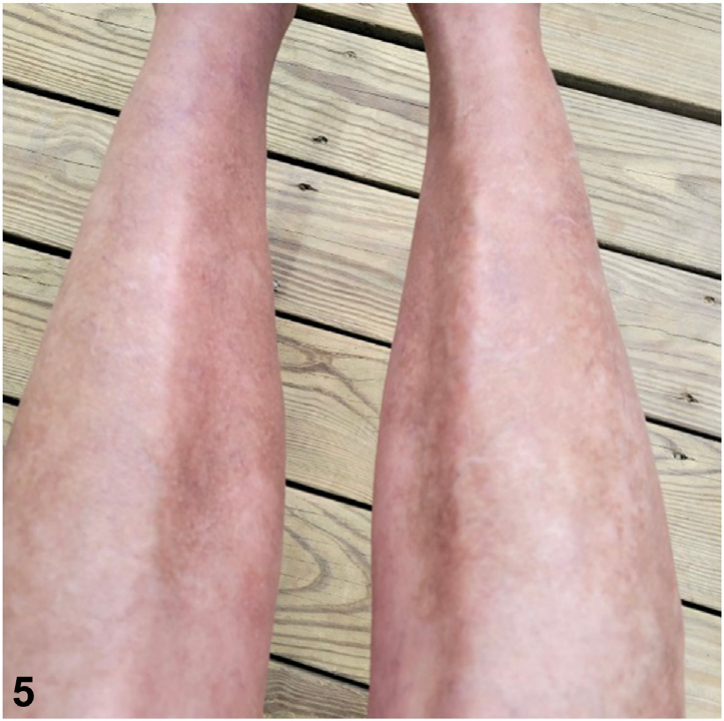

Further workup revealed an elevated glucagon level (1248 pg/mL) and diffuses hypoaminoacidemia. Magnetic resonance imaging (MRI) confirmed a pancreatic mass, and biopsy diagnosed a glucagonoma. The patient underwent distal pancreatectomy and splenectomy, with pathology confirming no lymph node involvement. Postoperative positron emission tomography/CT indicated no residual disease, and her symptoms resolved. Figs 4 and 5 below show resolution of the patient’s rash following surgical resection.

## Discussion

NME, the characteristic rash of glucagonoma, appears in up to 90% of cases and presents as painful, erythematous plaques with vesicles, bullae, or crusting.^1^ It must be distinguished from conditions such as acrodermatitis enteropathica, essential fatty acid deficiency, and pellagra.^2^^,^^3^ Although its exact mechanism is unclear, nutritional deficiencies, particularly fatty acids, are implicated.

Glucagonoma diagnosis requires tumor detection, elevated glucagon levels, and characteristic systemic findings such as rash, diabetes, or hypoaminoacidemia. Due to its rarity, diagnosis is often delayed, as in this case, where the rash was misattributed to atopic dermatitis, cellulitis, Stevens-Johnson syndrome, and UC-related dermatitis. The mass was not discovered on CT scan performed 4 months prior to her MRI either due to lower sensitivity compared to MRI or lack of significant mass size at the time.

Glucagonoma also induces a hypercoagulable state, increasing the risk of venous thromboembolism, as seen in this patient’s deep vein thrombosis and pulmonary embolism.^4^ Given that skin biopsy findings may be subtle or nonspecific, clinical-pathologic correlation is crucial. Recurring NME-like rashes despite treatment should prompt further investigation, including glucagon-level measurement. Early recognition and surgical resection improve outcomes, emphasizing the importance of considering glucagonoma in persistent, treatment-resistant dermatologic presentations.

## Conflicts of interest

None disclosed.
